# Congenital midline spinal hamartoma in an infant with DICER1 syndrome: A case report

**DOI:** 10.3389/fonc.2022.963768

**Published:** 2022-08-18

**Authors:** Rawan Hammad, Winnie Lo, Haiying Chen, Manohar Shroff, David Malkin, Anita Villani, Avram Denburg

**Affiliations:** ^1^ Division of Pediatric Hematology and Oncology, The Hospital for Sick Children, Toronto, ON, Canada; ^2^ Division of Hematology, Faculty of Medicine, King Abdulaziz University, Jeddah, Saudi Arabia; ^3^ Department of Pediatric Laboratory Medicine, The Hospital for Sick Children, Toronto, ON, Canada; ^4^ Department of Diagnostic Imaging, The Hospital for Sick Children, Toronto, ON, Canada

**Keywords:** congenital midline spinal hamartoma, DICER1, infant, case report, cancer predisposition

## Abstract

Congenital spinal hamartomas are rare benign tumors. They are mostly seen in infants and are typically asymptomatic at presentation. Spinal hamartomas have not been associated with any known cancer predisposition syndrome. DICER1 syndrome is a well-characterized cancer predisposition syndrome caused by a germline mutation in the *DICER1* gene, which shows variable expressivity. To our knowledge, spinal hamartoma has never been described in individuals with DICER1 syndrome. Here, we describe a rare association of congenital spinal hamartoma and DICER1 syndrome in a 5-week-old infant, with molecular findings suggestive of the implication of *DICER1* in the pathogenesis of this tumor.

## Introduction

Congenital midline spinal hamartomas are a rare but increasingly well-described clinical entity. They are benign tumors, characterized by a well-differentiated and mature overgrowth of local ectodermal and mesodermal elements, present in a disorganized manner (1). Spinal hamartomas typically present in infants and have been associated with skin dimples, cutaneous angiomata, subcutaneous masses or intact overlying skin. At presentation, most patients with spinal hamartomas are neurologically intact with no significant signs of spinal cord compression (2). Hamartomas have been described in patients with spinal dysraphism (3) and in most cases are not associated with any other congenital malformations or cancer predisposition syndromes.

DICER1 syndrome (OMIM #601200) is a rare tumor predisposition syndrome, with tendency to develop a wide range of benign and malignant tumors beginning in childhood and continuing into adulthood. DICER1 syndrome is caused by a germline pathogenic variant in the tumor suppressor gene, *DICER1* (4). Typical DICER1-associated tumors include pleuropulmonary blastoma, cystic nephroma, nasal chondromesenchymal hamartoma (NCMH), thyroid nodules, and ovarian Sertoli-Leydig cell tumor (5). Central nervous system (CNS) manifestations of DICER1 syndrome include pituitary blastoma, pineoblastoma, ciliary body medulloepithelioma, Embryonal tumor with multilayered rosettes (ETMR)-like infantile cerebellar tumor and primary DICER1 CNS-sarcoma (6). To our knowledge, spinal hamartomas have never been described in patients with DICER1 syndrome.

Herein, we present the case of a 5-week-old infant with an extremely atypical presentation of congenital midline spinal hamartoma of the cervical spine, who was found to harbor a pathogenic germline *DICER1* variant and a second somatic hit in the spinal hamartoma.

## Case presentation

**Figure 1:**
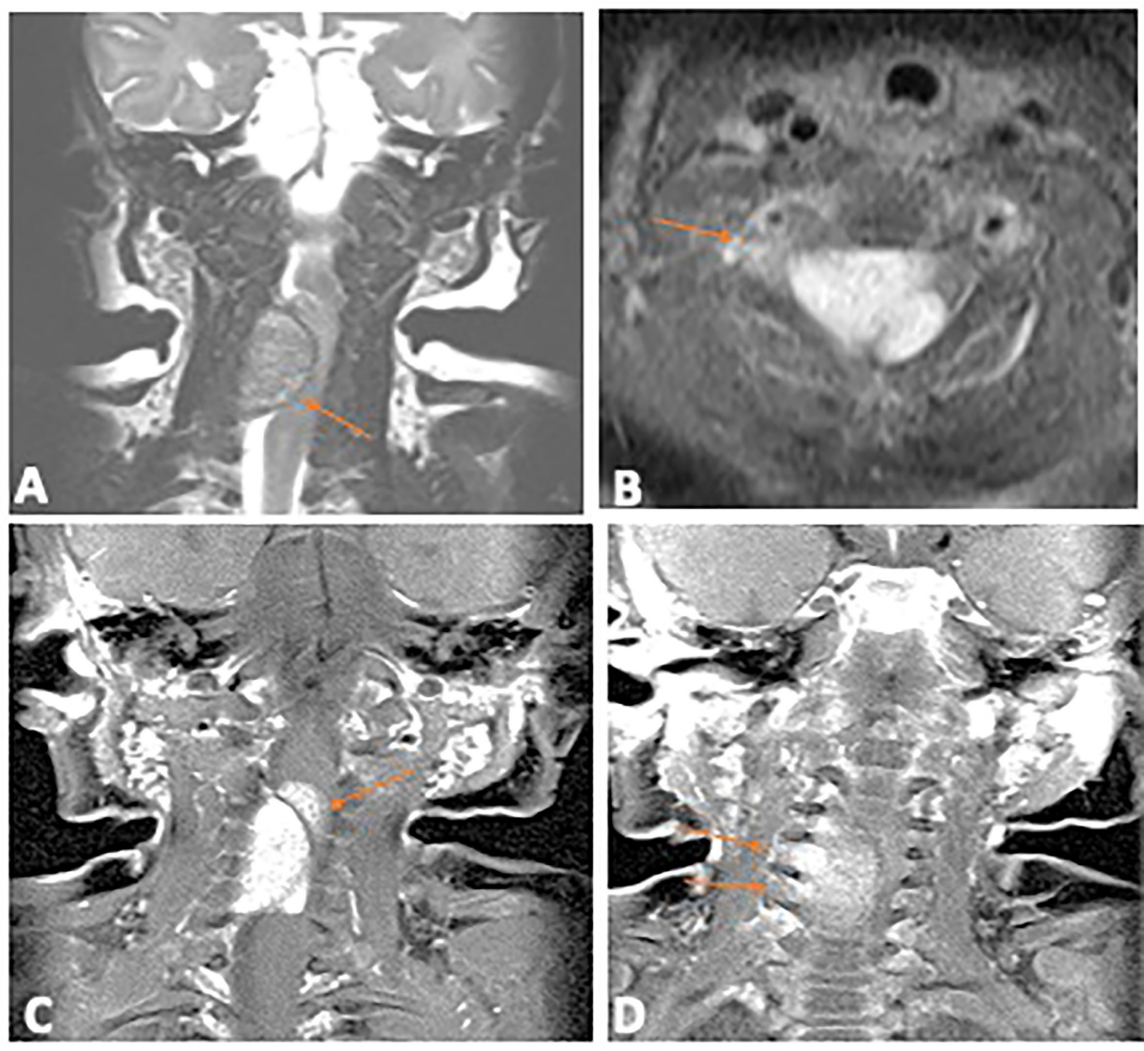
Diagnostic Magnetic Resonance Imaging of the primary spinal hamartoma. **(A)** Coronal T2 image of the cervical spine shows an intraspinal extramedullary mass (arrow) which is iso to hypointense, compressing the cord. **(B)** Contrast enhanced axial T1 image with fat saturation showing the enhancing intraspinal mass (arrow); **(C)** Coronal contrast enhanced T1 image with fat saturation showing the lobulated intraspinal component (arrow) which infiltrated into the cord. **(D)** Coronal contrast enhanced T1 image, arrows show extension through multiple neural foramina on the right.

A 5-week-old full-term female infant presented with progressive bilateral upper limb weakness and diminished spontaneous movements beginning at the age of 1 week. Her perinatal history was unremarkable, with no history of traumatic delivery and a normal physical exam at birth. At presentation, there was bilateral upper limb flaccid hypotonia on the right more than the left, absent grasp reflex in both hands, absent bilateral upper limb deep tendon reflexes and loss of upper limb sensation bilaterally. Magnetic resonance imaging (MRI) of the brain and spine revealed a cervical spine extramedullary lesion extending from C2-C6 with extension through several neuronal foramina on the right (**Figure 1**) and severe compression of the spinal cord.

Based on her age and radiographic features, neuroblastoma with cord compression was suspected. Staging Computed tomography (CT) of the neck, chest abdomen and pelvis were unremarkable. Her urine catecholamines (HVA and VMA) were both within normal limits on two occasions. She received a single cycle of carboplatin and etoposide as well as dexamethasone on an emergent basis. The patient continued to demonstrate symptomatic cord compression, so a decision was made to undergo surgical resection of the lesion to protect her cord from ongoing damage and obtain a histologic diagnosis.

**Figure 2:**
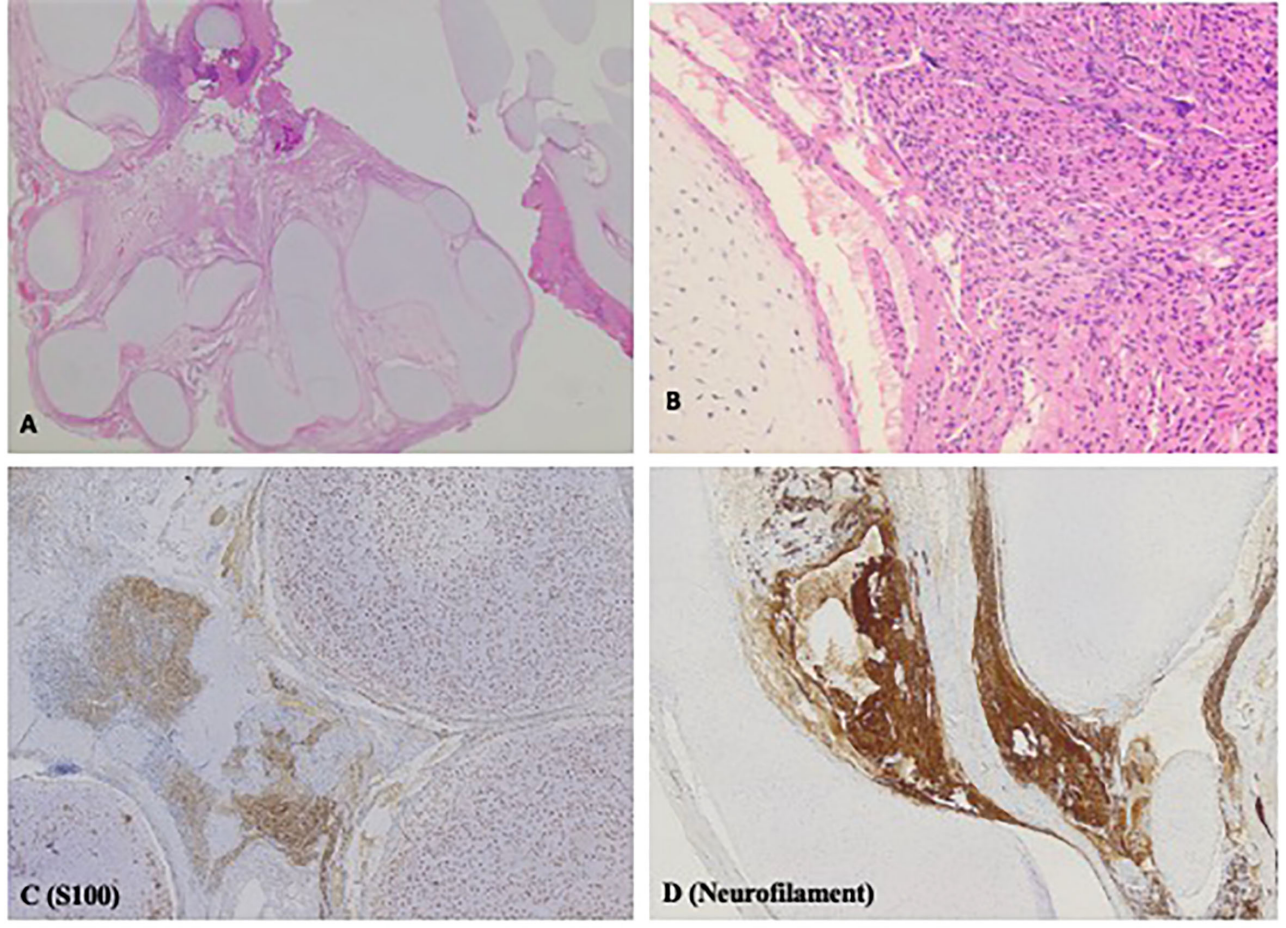
H&E and Immunohistochemistry (IHC) stains displaying histopathologic features of spinal hamartoma. **(A)** H&E stain; original magnification x12.5. Irregular lobules of hyaline cartilage, with loose fibrous stroma in between. Irregular nerve-like tissue is found in the loose fibrous stroma **(B)** H&E stain; original magnification x200. High power view of the cartilage and nerve-like tissue. **(C)** IHC stain for S100; original magnification x 50. Both the cartilage and the nerve-like tissue are positive for S100. **(D)** IHC stain for neurofilament; original magnification x50. The nerve-like tissue is positive for neurofilament.

Intra-operatively, a well circumscribed cartilaginous tumor was identified on the right side of the dura. A gross total resection of the extradural component was achieved and a small intradural residual tumor was left as it was adherent to the nerve root. Pathologic examination revealed a benign lesion with similar features in the intraspinal, extradural and intradural components. The lesion showed anastomosing islands of hyaline cartilage with loose fibrous stroma (including nerve-like tissue) in between (**Figures 2A, B**). Immunohistochemistry (IHC) stains of the nerve-like tissue were positive for neurofilament and S100, consistent with nerve differentiation (**Figures 2C, D**). IHC stains for glial fibrillary acidic protein (GFAP), pan-cytokeratin and desmin were negative. Overall, the features favor a diagnosis of spinal hamartoma.

Postoperatively, the patient developed respiratory failure secondary to accessory muscle weakness requiring ventilation and eventually a tracheostomy. Post-operative MRI showed two areas of extradural enhancement in the right aspect of the spinal canal suggestive of residual tumor.

Her family history revealed non-consanguineous parents of European descent. The mother had developed thyroid nodules at age 16 years and an ovarian Sertoli-Leydig cell tumor which was diagnosed and resected at age 30 years, following the diagnosis of our patient’s spinal hamartoma. There was no other family history of cancers or cancer predisposition in the family.

The evolution of this family’s cancer history prompted further evaluation. A salivary sample was obtained from the mother for germline *DICER1* sequencing using an external *DICER1* DNA sequencing panel (Prevention Genetics, Marshfield, WI). Results revealed a heterozygous pathogenic variant in the *DICER1* gene designated c.3118_3119insCA (p.IIe1040Thrfs*27), which is predicted to result in a frameshift and premature translational stop site, expected to result in an absent or disrupted protein product. This variant was reported as pathogenic given its predicted functional consequence and has not been previously reported in the literature nor in population controls (gnomAD). There was no tumor specimen available from the mother for sequencing.

Following the mother’s confirmed germline *DICER1* finding, the patient was enrolled in our institutional precision oncology program KiCS (SickKids Cancer Sequencing) Program, through which germline and tumor samples were sequenced using a clinically validated DNA cancer sequencing panel (supplementary material). The same heterozygous germline frameshift variant in the *DICER1* gene was detected in the patient who at that point was 3 years of age.

Sequence analysis of the patient’s tumor revealed a second missense variant in the *DICER1* gene, namely, c.5113G>A (p.Glu1705Lys). This is a somatic hot spot variant and is frequently identified somatically in DICER1 associated tumors such as Sertoli–Leydig cell tumor and pleuropulmonary blastoma (7, 8). Somatic hot spot missense variants in *DICER1* are clustered in the RNase IIIb domain and functional studies have shown that they lead to defective cleavage of 5p-derived miRNAs from the pre-miRNA loop sequence and results in the retention of the pre-miRNA loop sequence in *DICER1* mutant cancers (8). This was detected at a variant allele frequency (VAF) of 41.56%. No other gene variants were detected in the tumor. The tumor mutational burden was 0.33 mutations/Mb and copy number analysis showed a stable genome.

**Figure 3:**
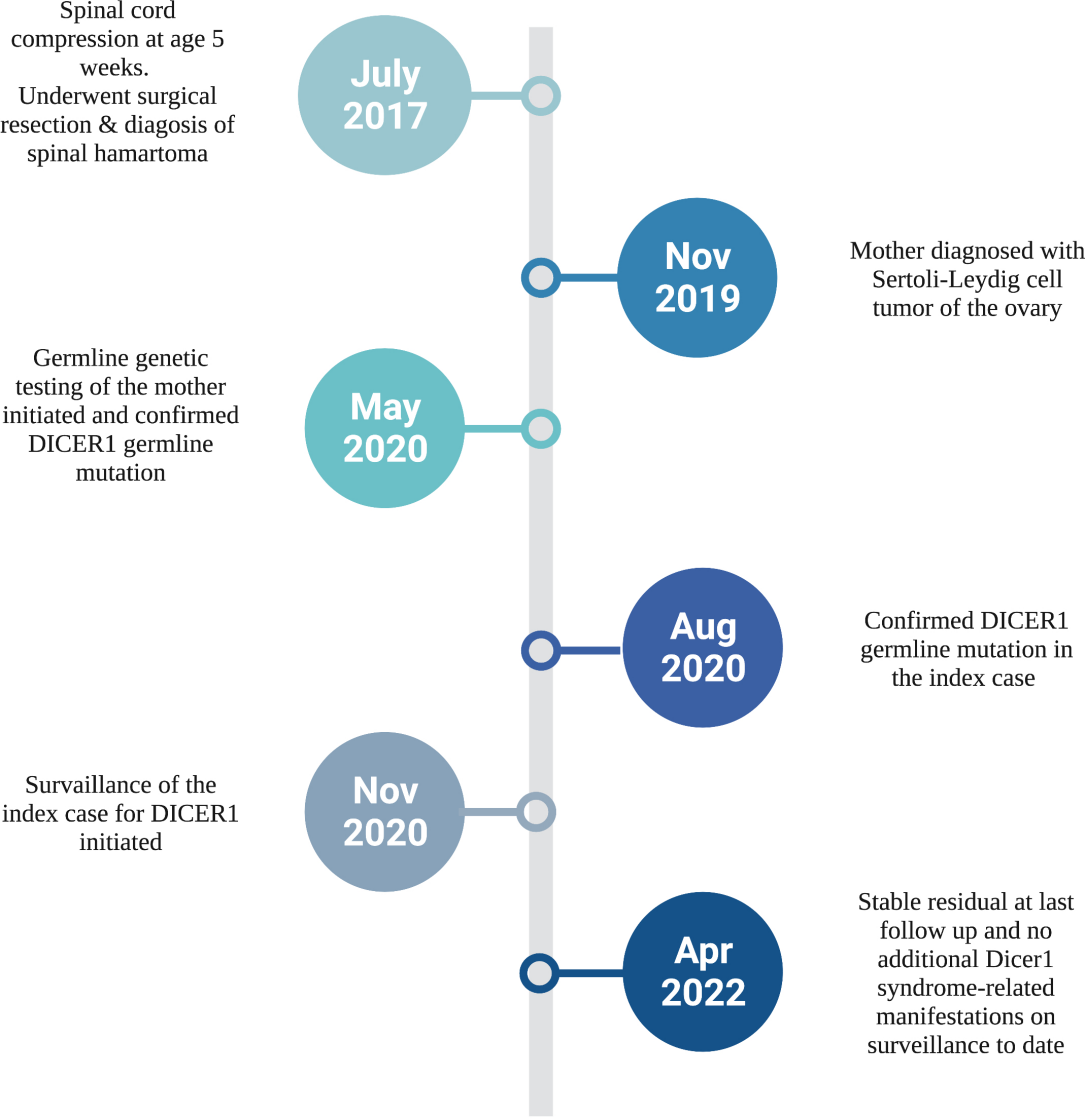
Diagnostic timeline of our infant with congenital spinal hamartoma and DICER1 Syndrome. Created with BioRender.com.

At her last follow up, at the age of four years, the patient remained tracheostomy-dependent but had shown marked interval improvement in her limb function. Serial surveillance MRIs of the spine showed stable residual with no signs of recurrence. Ongoing Surveillance with 6 monthly abdominal/pelvic ultrasound and chest X-ray and annual ophthalmologic assessment has not revealed any other DICER1-associated manifestations to date (9) (**Figure 3**).

## Discussion

In this report, we describe the first case of congenital midline spinal hamartoma in an infant with a germline *DICER1* pathogenic variant, an association not previously reported in the literature. The presence of a second somatic hotspot missense *DICER1* variant provides evidence that this tumor is a DICER-1 syndrome related manifestation in this patient.

DICER1 syndrome was first described by Priest et al. when several family members of children with pleuropulmonary blastema (PPB) were noted to develop other neoplasms (10). *DICER1* germline pathogenic variants were subsequently identified as an inciting genetic event in the development of this syndrome when families with PPB underwent germline DNA sequencing (11). Since the *DICER1* gene discovery, the list of associated benign and malignant neoplasms continues to expand (9). NCMH was the first and remains the most commonly reported type of hamartoma linked to *DICER1* syndrome (5). In 2014, Stewart et al. established the association in a case series in which 6/8 evaluable patients with PPB and NCMH were found to harbor a germline *DICER1* mutation; two of these were found to have an acquired somatic *DICER1* mutation (12). Further studies supported the association, and NCMH is now a well-established *DICER1-*associated tumor (5, 13, 14). Recently, Apellaniz-Ruiz described two patients with DICER1 syndrome and mesenchymal hamartoma of the liver (MHL). These authors hypothesized the direct implication of the *DICER1* gene in the pathogenesis of MHL, through dysregulation of miRNA expression, suggesting that MHL is a DICER1 phenotype (15). This variety of tumors supports the variable expressivity observed in DICER1 syndrome and indicates the possibility of further expansion of DICER1 associated neoplasms.

Our patient developed a congenital spinal hamartoma, which is an extremely rare benign, but locally aggressive, tumor of infancy and childhood. Intracranial hamartomas are commonly associated with neurofibromatosis type 1, consisting of neural-crest derived neurons, glial cells and Schwann cells (16). Congenital midline spinal hamartomas differ histopathologically as they are composed of mature native tissue which is present in a disordered manner (1). It was first reported as a distinct entity by Tibbs et al. in 1976, where the clinicopathologic characteristics of five infants with spinal hamartoma was described. The initial description noted the histologic distinctive features from teratomas, namely the presence of mesodermal elements, the well differentiated tissue and lack of malignant transformation in the former (17). The definition of spinal hamartomas was later refined to the presence of disorganized mature local tissue (1, 18). Since its early description, only a few case series and case reports have been reported in the literature, with the majority describing it as an asymptomatic, benign lesion lacking the potential for malignant transformation (2, 3, 19, 20). The pathogenesis of these hamartomas is unknown and has not been molecularly characterized.

*DICER1* is a tumor suppressor gene which encodes an RNase III-family endonuclease responsible for cleavage of precursor microRNA into active miRNA (21). Tumorigenesis in DICER1 syndrome is typically caused by a loss of function germline variant and a second somatic missense variant in one of the “hotspot” codons within the RNase IIIb domain (4). Functional studies of these somatic *DICER1* RNase IIIb domain missense variants have shown that they lead to defective cleavage of 5p-derived miRNAs from the pre-miRNA loop sequence resulting in the retention of pre-miRNA loop sequence in *DICER1* mutant cancers (8). This is consistent with the two-hit hypothesis implicated in the development of cancers in most patients with DICER1 syndrome (9). Our patient’s *DICER1* germline pathogenic variant was a c.3118_3119insCA frameshift. The second hit identified in our patient’s tumor was a missense mutation in the *DICER1* gene (c.5113G>A (p.Glu1705Lys)). This variant is one of the hot spot mutations in *DICER1* and is frequently identified somatically in Sertoli–Leydig cell tumors, pleuropulmonary blastomas and other cancer types. It is often found with a loss of function germline *DICER1* variant in patients with DICER1 tumor predisposition syndrome (7).

It is interesting to note that our patient’s spinal hamartoma is quite atypical in many ways. First, the hamartoma developed in the cervical spine, contrary to the thoracic and lumbosacral predominance that is generally described in the literature (1). Additionally, spinal hamartomas are usually asymptomatic, whereas our patient presented with significant symptomatic spinal cord compression, which has only been reported once previously (20). It is not clear if the pathogenesis and clinical behavior of this hamartoma is influenced by the *DICER1* drivers, differentiating its natural history from sporadic forms of spinal hamartoma.

In summary, *DICER1*-associated hamartoma has been described in the nasal cavity, liver and now spine. As described in this report, congenital spinal hamartoma is a newly associated, albeit rare manifestation of the *DICER1* syndrome phenotype.

## Data availability statement

The original contributions presented in the study are included in the article/**Supplementary Material**. Further inquiries can be directed to the corresponding author/s.

## Ethics statement

Ethical review and approval was not required for the study on human participants in accordance with the local legislation and institutional requirements. Written informed consent to participate in this study was provided by the participants’ legal guardian/next of kin. Written informed consent was obtained from the individual(s), and minor(s)’ legal guardian/next of kin, for the publication of any potentially identifiable images or data included in this article.

## Author contributions

RH, data acquisition and original draft preparation. WL, carried out molecular genetic studies and annotation. HC, pathologist provided histology images. MS, radiologist provided MRI images. DM, AV, and AD, conceptualization, writing- review and editing. All authors contributed to the article and approved the submitted version.

## Conflict of interest

The authors declare that the research was conducted in the absence of any commercial or financial relationships that could be construed as a potential conflict of interest.

## Publisher’s note

All claims expressed in this article are solely those of the authors and do not necessarily represent those of their affiliated organizations, or those of the publisher, the editors and the reviewers. Any product that may be evaluated in this article, or claim that may be made by its manufacturer, is not guaranteed or endorsed by the publisher.
