# Supplementary material for: Congenital midline spinal hamartoma in an infant with DICER1 syndrome: A case report
Source: Front Oncol. 2022 Aug 18;12:963768. doi: 10.3389/fonc.2022.963768 (PMC9433698; doi:10.3389/fonc.2022.963768)
Supplement: Supplementary file 1 [file DataSheet_1.docx]

**Supplementary material**

**Figure S1:** 864 Genes included on KiCS Comprehensive Cancer Panel.

| ABCB1 | AXIN1 | CCND1 | COL2A1 | DSCAM | FAM46C | GABRG1 | HIST4H4 | KAT6A | MAP2K4 | MXRA5 | NUTM1 | POLD1 | RANBP17 | SETD2 | TAF15 | TRIP11 |
| --- | --- | --- | --- | --- | --- | --- | --- | --- | --- | --- | --- | --- | --- | --- | --- | --- |
| ABCB11 | AXIN2 | CCND2 | COL5A1 | DST | FANCA | GALNT12 | HLA-A | KAT6B | MAP3K1 | MYB | ODC1 | POLE | RANBP2 | SETDB1 | TAF1L | TRRAP |
| ABI1 | B2M | CCND3 | COL7A1 | DYNC1H1 | FANCC | GALNT15 | HLA-B | KCNJ5 | MAP3K7 | MYC | OLIG2 | POLQ | RAP1GDS1 | SF3B1 | TAL1 | TSC1 |
| ABL1 | BAP1 | CCNE1 | COLEC12 | EBF1 | FANCD2 | GAS7 | HLF | KDM3B | MAPK1 | MYCL | OMD | POT1 | RARA | SFPQ | TAL2 | TSC2 |
| ABL2 | BARD1 | CD274 | COX6C | ECT2L | FANCE | GATA1 | HMGA1 | KDM4C | MAPK8 | MYCN | OR4A16 | POTEF | RB1 | SGK1 | TBL1XR1 | TSHR |
| ACKR3 | BAZ2A | CD70 | CREB1 | EED | FANCF | GATA2 | HMGA2 | KDM5A | MAPK8IP1 | MYD88 | OTOF | POU2AF1 | RBL1 | SH2B3 | TBX18 | TSHZ3 |
| ACSL3 | BCL10 | CD74 | CREB3L1 | EGFR | FANCG | GATA3 | HNF1A | KDM5C | MARK1 | MYH1 | OTX2 | POU2F2 | RBM10 | SH2D1A | TBX22 | TTL |
| ACSL6 | BCL11A | CD79A | CREB3L2 | EGR3 | FAP | GEN1 | HNRNPA2B1 | KDM6A | MARK4 | MYH11 | P2RY8 | POU5F1 | RBM15 | SH3GL1 | TBX3 | U2AF1 |
| ACVR1 | BCL11B | CD79B | CREBBP | EIF4A2 | FAS | GFI1B | HNRNPR | KDR | MAST4 | MYH2 | PAFAH1B2 | PPARG | RECQL4 | SIN3A | TCEA1 | UBR5 |
| ACVR1B | BCL2 | CDC6 | CRLF2 | ELF3 | FASN | GMPS | HOOK3 | KDSR | MATK | MYH9 | PAK3 | PPM1D | REL | SLC26A3 | TCEB1 | UHRF2 |
| ACVR2A | BCL2L1 | CDC73 | CRTC1 | ELF4 | FAT1 | GNA11 | HOXA11 | KEAP1 | MAX | NACA | PALB2 | PPP1R3A | RELA | SLC34A2 | TCF12 | UNC13C |
| ADAMTS20 | BCL3 | CDH1 | CRTC3 | ELK4 | FBN2 | GNA13 | HOXA13 | KIAA1549 | MBD1 | NAV1 | PARK2 | PPP2R1A | RELN | SLC45A3 | TCF3 | USP6 |
| AFF1 | BCL6 | CDH10 | CSF1R | ELL | FBXO11 | GNAQ | HOXA9 | KIF5B | MCL1 | NAV3 | PARP2 | PPP6C | RET | SLITRK5 | TCF7 | USP7 |
| AFF3 | BCL7A | CDH11 | CSF2RA | ELN | FBXW7 | GNAS | HOXC11 | KIF7 | MDC1 | NBN | PATZ1 | PRCC | RHBDF2 | SMAD2 | TCF7L2 | USP9X |
| AFF4 | BCL9 | CDH20 | CSF3R | EML4 | FCGR2B | GOLGA5 | HOXC13 | KIT | MDM2 | NCAPD3 | PAX3 | PRDM1 | RHEB | SMAD4 | TCL1A | VANGL1 |
| AJUBA | BCL9L | CDK12 | CSMD3 | EP300 | FCRL2 | GOPC | HOXD11 | KLF4 | MDM4 | NCKIPSD | PAX5 | PRDM16 | RHOA | SMARCA4 | TERT | VHL |
| AKAP9 | BCLAF1 | CDK4 | CTCF | EP400 | FCRL4 | GPAM | HOXD13 | KLF6 | MECOM | NCOA1 | PAX7 | PRDM2 | RHOH | SMARCB1 | TET1 | VTI1A |
| AKT1 | BCOR | CDK6 | CTNNA1 | EPB41L3 | FES | GPC3 | HRAS | KLK2 | MED12 | NCOA2 | PAX8 | PRDM9 | RIMS2 | SMARCE1 | TET2 | WAS |
| AKT2 | BCR | CDK8 | CTNNA2 | EPCAM | FEV | GPHN | HSP90AA1 | KMT2A | MED13 | NCOA3 | PBRM1 | PREX2 | RMI2 | SMC1A | TFE3 | WDFY3 |
| AKT3 | BHMT2 | CDKN1B | CTNNB1 | EPHA2 | FGFR1 | GPS2 | HSP90AB1 | KMT2C | MEN1 | NCOA4 | PBX1 | PRF1 | RNASEL | SMC3 | TFEB | WDFY4 |
| ALDH2 | BIRC3 | CDKN2A | CTNND1 | EPHA3 | FGFR1OP | GREM1 | HSPB8 | KMT2D | MET | NCOR1 | PCBP1 | PRKAG2 | RNF213 | SMO | TFG | WHSC1 |
| ALK | BLM | CDKN2B | CTTN | EPHA6 | FGFR2 | GRM3 | HUWE1 | KRAS | MGA | NCOR2 | PCDH11X | PRKAR1A | RNF217 | SOCS1 | TFPT | WHSC1L1 |
| ALPK2 | BMPR1A | CDKN2C | CUL3 | EPHA7 | FGFR3 | GRM8 | ICK | KTN1 | MGMT | NDRG1 | PCDH15 | PRKCD | RNF43 | SOS1 | TFRC | WIF1 |
| AMER1 | BRAF | CDX2 | CUL4B | EPHB1 | FGFR4 | GUCY1A2 | IDH1 | LASP1 | MITF | NEDD4L | PCDHGB3 | PRKDC | ROS1 | SOX17 | TGFBR2 | WRN |
| APAF1 | BRCA1 | CEBPA | CUX1 | EPHB4 | FH | H2AFY | IDH2 | LCK | MKL1 | NEK8 | PCM1 | PRLR | RPL10 | SOX2 | THBS1 | WT1 |
| APC | BRCA2 | CENPF | CYLD | EPHB6 | FHIT | H3F3A | IFIT3 | LCP1 | MLF1 | NF1 | PCSK7 | PRRC2A | RPL22 | SOX9 | THRAP3 | WWTR1 |
| ARAF | BRD3 | CHCHD7 | CYP1A1 | EPPK1 | FIP1L1 | H3F3B | IGF1R | LEPROT | MLH1 | NF2 | PDCD1LG2 | PRRX1 | RPL5 | SPECC1 | TLR4 | XIRP2 |
| ARHGAP26 | BRD4 | CHD1 | CYP21A2 | EPS15 | FLCN | H3F3C | IGF2 | LHFP | MLLT1 | NFE2 | PDE4DIP | PRSS58 | RPN1 | SPOP | TLX1 | XPA |
| ARHGAP35 | BRIP1 | CHD3 | DAB2IP | ERBB2 | FLG | HDAC6 | IGF2R | LIFR | MLLT10 | NFE2L2 | PDGFB | PRX | RPS15 | SPTAN1 | TLX3 | XPC |
| ARHGEF12 | BRWD3 | CHD4 | DACH2 | ERBB3 | FLI1 | HDAC9 | IKBKB | LIN28B | MLLT11 | NFIB | PDGFRA | PSIP1 | RPS2 | SRC | TMEM127 | XPO1 |
| ARID1A | BTG1 | CHD6 | DAXX | ERBB4 | FLNA | HERPUD1 | IKZF1 | LMO1 | MLLT3 | NFKB1 | PDGFRB | PTCH1 | RPS6KA2 | SRCAP | TMPRSS2 | XRCC2 |
| ARID1B | BTK | CHD7 | DCC | ERC1 | FLT1 | HEY1 | IL2 | LMO2 | MLLT4 | NFKB2 | PER1 | PTEN | RRM1 | SRGAP3 | TNF | YWHAE |
| ARID2 | BUB1B | CHD8 | DDB2 | ERC2 | FLT3 | HFE | IL21R | LPP | MLLT6 | NIN | PFKP | PTGS2 | RSPO2 | SRSF2 | TNFAIP3 | YY1 |
| ARID5B | C15orf65 | CHEK1 | DDIT3 | ERCC1 | FLT4 | HIP1 | IL3 | LRFN5 | MMP2 | NKX2-1 | PHF20 | PTPN11 | RSRC1 | SRSF3 | TNFRSF14 | ZBTB16 |
| ARNT | C2orf44 | CHEK2 | DDX10 | ERCC2 | FMN2 | HIST1H1B | IL6ST | LRIG3 | MN1 | NLRP5 | PHF6 | PTPN13 | RUNX1 | SS18 | TNFRSF17 | ZEB2 |
| ARTN | CACNA1D | CHIC2 | DDX3X | ERCC3 | FN1 | HIST1H1C | IL7R | LRP1B | MNX1 | NONO | PHIP | PTPRC | RUNX1T1 | SS18L1 | TNK2 | ZFHX3 |
| ASPSCR1 | CALCR | CHN1 | DDX5 | ERCC4 | FNBP1 | HIST1H1E | ING1 | LRP2 | MPL | NOTCH1 | PHOX2B | PTPRD | RXRA | SSX1 | TNN | ZFP36L1 |
| ASTN1 | CALR | CIC | DDX6 | ERCC5 | FOSL2 | HIST1H2BD | ING4 | LRRC7 | MPO | NOTCH2 | PICALM | PTPRT | SAMD9 | STAG2 | TNR | ZFP36L2 |
| ASXL1 | CAMTA1 | CIITA | DDX60 | ERCC6 | FOXA1 | HIST1H4A | INTS1 | LRRK1 | MRE11A | NOTCH4 | PIK3CA | QKI | SBDS | STAT3 | TOP1 | ZHX2 |
| ASXL2 | CANT1 | CLP1 | DEK | ERG | FOXA2 | HIST1H4B | IRF4 | LRRK2 | MSH2 | NPAP1 | PIK3CB | RAB11FIP1 | SDC4 | STAT5B | TP53 | ZMYM2 |
| ATF1 | CARD11 | CLSTN1 | DEPDC5 | ESR1 | FOXL2 | HIST1H4C | IRS2 | LRRTM4 | MSH6 | NPM1 | PIK3CG | RABEP1 | SDHA | STIL | TP53BP1 | ZMYM3 |
| ATF7IP | CARS | CLSTN2 | DICER1 | ETS1 | FOXO1 | HIST1H4D | IRX2 | LTK | MSI2 | NR4A3 | PIK3R1 | RAC1 | SDHAF2 | STK11 | TPM3 | ZMYM4 |
| ATIC | CASC5 | CLTC | DIS3 | ETV1 | FOXO3 | HIST1H4E | ITK | LYL1 | MSN | NRAS | PIK3R2 | RAD21 | SDHB | STK19 | TPM4 | ZMYND8 |
| ATM | CASP8 | CLTCL1 | DIS3L2 | ETV4 | FOXO4 | HIST1H4F | ITPKB | LYN | MTAP | NRP1 | PIM1 | RAD50 | SDHC | STK36 | TPO | ZNF331 |
| ATP10A | CBFA2T3 | CNBP | DKC1 | ETV5 | FOXP1 | HIST1H4H | JAK1 | MAF | MTCP1 | NSD1 | PKHD1 | RAD51 | SDHD | STK4 | TPR | ZNF384 |
| ATP1A1 | CBFB | CNOT3 | DNAH14 | ETV6 | FOXQ1 | HIST1H4I | JAK2 | MAFB | MTOR | NT5C2 | PLAG1 | RAD51B | SEPT5 | SUFU | TRAF3 | ZNF521 |
| ATP2B3 | CBL | CNTN5 | DNM2 | EWSR1 | FSCB | HIST1H4J | JAK3 | MALT1 | MUC1 | NTRK1 | PLCG1 | RAD51C | SEPT6 | SUZ12 | TRAF7 | ZNF91 |
| ATRX | CBLB | CNTRL | DNMT3A | EXT1 | FSHR | HIST1H4K | JAZF1 | MAML2 | MUC16 | NTRK3 | PLCG2 | RAD51D | SEPT9 | SYK | TRAT1 | ZRSR2 |
| AURKA | CBLC | COL11A1 | DOCK2 | EXT2 | FSTL3 | HIST1H4L | JMJD1C | MAP1B | MUC17 | NUMA1 | PML | RAF1 | SERPINE1 | SYNE1 | TRIM24 |  |
| AURKAIP1 | CCDC6 | COL19A1 | DPP10 | EZH2 | FUBP1 | HIST2H4A | JUN | MAP2K1 | MUC4 | NUP214 | PMS1 | RAG1 | SET | SYNGAP1 | TRIM27 |  |
| AURKB | CCNB1IP1 | COL1A1 | DPYD | EZR | FUS | HIST2H4B | KALRN | MAP2K2 | MUTYH | NUP98 | PMS2 | RALGDS | SETBP1 | TAF1 | TRIM33 |  |

Next generation sequencing was done using Agilent SureSelect capture followed by paired-end sequencing of the coding and splice site regions using the Illumina sequencing platform. Variant calls are generated using Genome Analysis Tool Kit (GATK) after read alignment Burrows-Wheeler Aligner (BWA). Somatic variants are called against the matched germline sample by tumor-normal subtractive analysis.
